# Identification of QTLs for Reduced Susceptibility to Rose Rosette Disease in Diploid Roses

**DOI:** 10.3390/pathogens11060660

**Published:** 2022-06-08

**Authors:** Ellen L. Young, Jeekin Lau, Nolan B. Bentley, Zena Rawandoozi, Sara Collins, Mark T. Windham, Patricia E. Klein, David H. Byrne, Oscar Riera-Lizarazu

**Affiliations:** 1 Department of Horticultural Sciences, Texas A&M University, College Station, TX 77843, USA; eroundey@gmail.com (E.L.Y.); jzl0026@tamu.edu (J.L.); zena.rawandoozi@ag.tamu.edu (Z.R.); patricia.klein@ag.tamu.edu (P.E.K.); david.byrne@ag.tamu.edu (D.H.B.); 2 Department of Integrative Biology, University of Texas at Austin, Austin, TX 78705, USA; nolanbentley@utexas.edu; 3 Department of Entomology and Plant Pathology, Institute of Agriculture, University of Tennessee, Knoxville, TN 37996, USA; scolli41@utk.edu (S.C.); mwindham@utk.edu (M.T.W.)

**Keywords:** Rosa, emaravirus, QTL, virus resistance, plant breeding

## Abstract

Resistance to rose rosette disease (RRD), a fatal disease of roses (*Rosa* spp.), is a high priority for rose breeding. As RRD resistance is time-consuming to phenotype, the identification of genetic markers for resistance could expedite breeding efforts. However, little is known about the genetics of RRD resistance. Therefore, we performed a quantitative trait locus (QTL) analysis on a set of inter-related diploid rose populations phenotyped for RRD resistance and identified four QTLs. Two QTLs were found in multiple years. The most consistent QTL is *qRRV_TX2WSE_ch5*, which explains approximately 20% and 40% of the phenotypic variation in virus quantity and severity of RRD symptoms, respectively. The second, a QTL on chromosome 1, *qRRD_TX2WSE_ch1*, accounts for approximately 16% of the phenotypic variation for severity. Finally, a third QTL on chromosome 3 was identified only in the multiyear analysis, and a fourth on chromosome 6 was identified in data from one year only. In addition, haplotypes associated with significant changes in virus quantity and severity were identified for *qRRV_TX2WSE_ch5* and *qRRD_TX2WSE_ch1*. This research represents the first report of genetic determinants of resistance to RRD. In addition, marker trait associations discovered here will enable better parental selection when breeding for RRD resistance and pave the way for marker-assisted selection for RRD resistance.

## 1. Introduction

Rose rosette disease (RRD) of roses (*Rosa* spp.), is endemic to North America and, unlike other rose diseases, is fatal for an infected plant [1]. Thus, RRD poses a major threat to the United States garden rose industry, which was valued at USD 168 million in 2019 [2]. RRD has also been reported in India [3], and while not yet reported in Europe, rose plants imported from North America and India are subject to regulations to prevent the introduction of RRD to Europe [4]. The disease is spread by the eriophyid mite *Phyllocoptes fructiphilus* Keifer [5,6]. The classic symptom is a witches’ broom or rosette growth, but symptoms can vary. Plants can also be asymptomatic for multiple years while testing positive for the presence of virus [7]. Unfortunately, no effective treatments exist, and as the disease may take several years to kill a plant, the current control recommendation is to remove infected plants to prevent the spread of the disease to other plants [7,8]. After three to four years of infection, RRD is generally fatal [7]. 

RRD is caused by the *Rose rosette emaravirus* (RRV), a membrane-bound negative-sense RNA virus in the family *Fimoviridae* [9,10,11]. While initially only four RNA segments were identified [9], RRV is now known to have seven segments [10], similar to other emaraviruses, which have five to 10 genome segments [11]. In RRV, RNA 1 encodes the RNA-dependent RNA polymerase; RNA 2, a glycoprotein; RNA 3, the nucleocapsid; RNA 4, the movement protein; and RNAs 5–7 are of unconfirmed function. Inoculation with RNAs 1–4 has been shown to be sufficient for systemic infection of *Nicotiana benthamiana* [12]. While mutations in the viral genome have been identified, the viral population is relatively homogenous across the United States [13]. Diseases caused by emaraviruses, including RRV, have been known for some time, but only in the past two decades have emaraviruses begun to be sequenced and characterized [11,14], and resistance to most emaraviruses has yet to be well-understood.

Due to the deadly nature of the disease and the lack of adequate control methods, roses resistant to RRD are highly desirable. Rose breeding is complicated, however, by a relatively long generation time and by the multispecies origin of most rose cultivars [15], most of which are diploid (2n = 2x = 14), triploid, or tetraploid [16]. Breeding for RRD resistance is particularly challenging since it is estimated that over 90% of cultivars are susceptible. A few rose species (among them the diploid species *Rosa setigera* Michaux), species hybrids, and a few cultivars may have some degree of resistance or reduced susceptibility to RRD [15]. Multiyear field trials are needed to accurately determine resistance levels due to the possibility of long virus latency periods and inconsistent disease pressure producing the appearance of resistance. Thus, RRD resistance is a good candidate for marker-assisted selection (MAS). 

The objective of this study was to identify quantitative trait loci (QTLs) for RRD resistance in diploid roses to improve the understanding of the genetic basis of RRD resistance and enable MAS. If the QTL analysis identifies markers that are applicable to a broad range of germplasm, MAS could be used for parental selection, designing crosses, tracking QTLs, and perhaps eliminating the need for field trials in screening for RRD resistance, thereby accelerating the breeding of resistant cultivars. In this study, four QTLs for reduced susceptibility were identified in diploid roses assessed for RRD susceptibility over three years, the most notable of which explains approximately 40% of the phenotypic variance for RRD severity.

## 2. Results

### 2.1. Phenotypic Data Analysis

**Figure 1:**
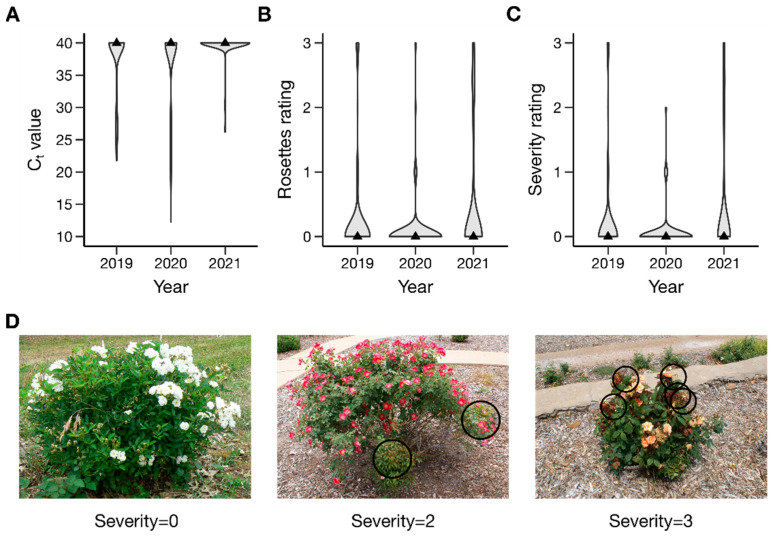
Distribution of rose rosette disease. (**A**) C_t_ value; (**B**) rosette ratings; (**C**) severity ratings in 2019, 2020, and 2021 across diploid rose families grown in Crossville, Tennessee; and (**D**) examples of the RRD severity scale with symptoms indicated by black circles. In violin plots, width of each shaded portion reflects the proportion of samples in that area. Black triangles indicate the median. Within each trait for a given year, the median, first interquartile, and third interquartile were equivalent; thus, only the median was plotted. Samples that were negative for RRV as determined by qRT-PCR were assigned a C_t_ value of 40. Rosettes were scored on a scale of 0–3, where 0 = no rosettes, 1 = one rosette, 2 = two rosettes, and 3 = three or more rosettes. Severity was rated on a scale of 0–3, where 0 = no symptoms, 1 = one shoot with symptoms, 2 = two shoots with symptoms, and 3 = three or more shoots with symptoms. Photographs of infected plants courtesy of Jennifer Olson, Oklahoma State University.

From 2019 to 2021, inter-related diploid rose populations were assessed for RRD resistance via qRT-PCR for virus presence and visually for the number of rosettes and severity of symptoms. Cycle threshold (C_t_) values obtained from positive virus tests were used to approximate quantification of the virus and ranged from 21.8 to 34.4, and rosette and severity scores both ranged from 0 to 3. In all years and overall, the data were skewed towards high C_t_ values, indicating low viral presence, and low rosette and severity scores, with a minority of genotypes having the virus detectable by qRT-PCR or visible symptoms of RRD (Figure 1, Table S1). In 2019, 47 of 248 genotypes (19%) tested positive for RRV, and only 39 of 248 genotypes (16%) had visible symptoms. In subsequent years, only genotypes not positive previously were tested for virus presence, but all were assessed visually for RRD. In 2020, 41 of the 201 genotypes that were negative in 2019 tested positive for RRV and 19 of the total number of genotypes had visible symptoms. This decline in visible symptoms between years may be partly due to the routine pruning of plants received in the spring which removed rosettes. In 2021, only two additional genotypes tested positive for RRV, and 47 genotypes had visible symptoms. At the end of three years, 90 of 248 genotypes (36%) tested positive for RRV, and 82 of 248 (33%) had shown visible symptoms at some point. Number of rosettes and severity were highly correlated (ρ > 0.99, *p* < 0.05) and had a moderate negative correlation with C_t_ value over years. Both number of rosettes and severity had moderate broad-sense heritability (H^2^ = 0.44 and 0.45, respectively).

### 2.2. Linkage Map

**Figure 2:**
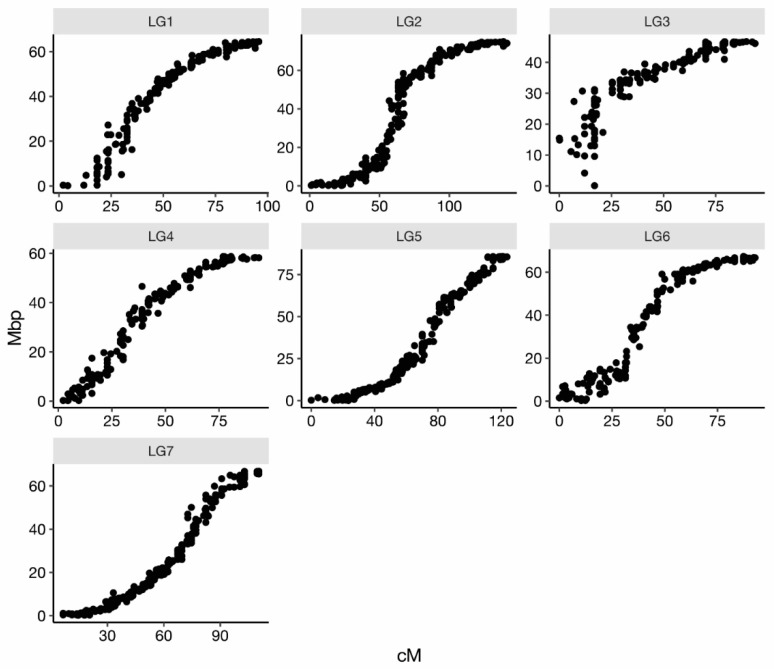
Alignment of the consensus map developed for three diploid rose populations with the *Rosa chinensis* genome assembly of Hibrand Saint-Oyant et al. [17] per linkage group (LG). Centimorgans (cM) are plotted on the *x*-axis and physical position (Mbp) on the *y*-axis.

Linkage maps were generated for each of the three largest populations (J14-3xPH, T7-20xSE, and T7-30xSE), and these maps were combined to create a consensus map. The individual population maps contained between 5239 and 9408 markers; 1583 of these were common between the three populations (Table S2). The consensus map had 2677 markers clustered in seven linkage groups and a total length of 758.2 cM, resulting in 1.5 unique positions/cM. Overall, the map had good coverage of the rose genome as well as good alignment with the *Rosa chinensis* genome of Hibrand Saint-Oyant et al. [17] (Figure 2).

### 2.3. QTL Analysis

FlexQTL™ (Wageningen University and Research, Wageningen, The Netherlands) was used for the QTL analysis. FlexQTL™ implements a Bayesian approach to QTL analysis that exploits the inter-relatedness of populations to improve the power and accuracy of QTL detection [18]. Each analysis begins with a different seed, leading to potentially different results for a single trait; therefore, the analysis was run four times for each trait (C_t_ value, 2019 severity, 2020 severity, 2021 severity, and overall severity) to ensure QTL reproducibility. Thus, the values reported here for heritability, phenotypic variance explained, and QTL position resulted from multiple runs for each trait. Two times the natural log of the Bayes factor (2lnBF) was used to determine QTL significance, with 2lnBF > 2 indicating positive evidence for a QTL. An additive model was used for severity and a mixed model for C_t_ value. Estimates of heritability by FlexQTL™ are restricted to the model used; thus, the program was used to estimate the broad-sense heritability (H^2^) of C_t_ value and narrow-sense heritability (h^2^) for RRD severity overall and individual years. H^2^ for C_t_ value ranged between 0.18 and 0.27 in contrast to the REML-estimated H^2^ of 0.45 for severity. H^2^ for severity overall, as estimated by FlexQTL™, was approximately 0.31 and ranged between 0.1 and 0.2 in individual years. 

**Figure 3:**
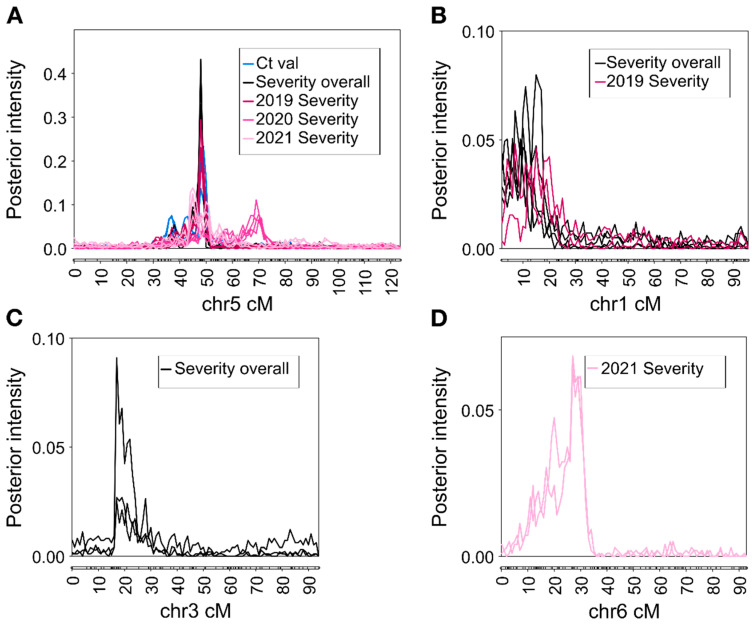
Positions and posterior intensities of identified QTL for (**A**) RRD C_t_ value and severity on chromosome 5, (**B**) RRD severity on chromosome 1, (**C**) RRD severity on chromosome 3, and (**D**) severity in 2021 on chromosome 6 in diploid rose populations. Multiple lines for the same color reflect the results of individual runs in FlexQTL™. Figure constructed with MapChart v2.32 (Wageningen University and Research, Wageningen, The Netherlands).

A strong to decisive QTL (2lnBF > 5 or 2lnBF > 10, respectively) was detected on chromosome 5 in all runs (Figure 3A) for C_t_ value and severity; no QTLs with 2lnBF > 2 on other chromosomes were consistently detected for C_t_ value (Table 1). The QTL peak, defined as the mode of the QTL, was located between 46 and 51 cM and was most frequently located at 49 cM (corresponding to a position of approximately 10 Mbp in the *Rosa chinensis* genome assembly reported by Hibrand Saint-Oyant et al. [17]). An exception was the results of 2020 severity, for which the peak was at 68–72 cM and which, in two runs, the QTL interval did not include the consensus position. Due to these inconsistencies, the single-year 2020 severity results were not pursued further though the 2020 data were still included in the overall analysis. The 2021 severity analyses indicated the presence of a second QTL on chromosome 5; however, the location was highly inconsistent between runs, and in some runs, the interval could not be defined using the standards established beforehand (see materials and methods). The QTL with a peak at 49 cM was assumed to be the same for both C_t_ value and severity and will be referred to as *qRRV_TX2WSE_ch5*. The consensus interval for *qRRV_TX2WSE_ch5*, defined as the interval common to all runs except the single-year runs for 2020 severity, was approximately 4.3 Mbp in size (6.8–11.1 Mbp). This interval in the *R. chinensis* genome assembly of Hibrand Saint-Oyant et al. [17] contains ~545 annotated genes. Of these, there were six NBS-LRR-class disease-resistance genes, two other genes associated with disease resistance, one gene for a eukaryotic initiation factor (eIF), four genes for transcription factors, and eleven ribosome-associated genes.

Three further QTLs for severity were also identified. A positive QTL (2lnBF > 2) for severity was detected on chromosome 1 in all runs for overall severity and two runs for 2019 severity (Figure 3B). This QTL will be referred to as *qRRD_TX2WSE_ch1*. The peak of the QTL was most often located between 8 and 16 cM (corresponding to roughly 0.3 and 4.8 Mbp, respectively). The QTL had a consensus interval approximately 12.1 Mbp in size (0.4–12.5 Mbp) and contains ~960 genes. These genes include 29 NBS-LRR-class disease-resistance genes, 44 disease-resistance genes of other types, 3 genes for transcription factors, and 19 ribosome-associated genes. A positive QTL for overall severity, *qRRD_TX2WSE_ch3*, was detected on chromosome 3 with a peak at 18 cM (approximately 27.8 Mbp). This QTL had a consensus interval approximately 24 Mbp in size (9.6–33.6 Mbp) and contains approximately 1900 genes. A strong QTL for 2021 severity, *qRRD_TX2WSE_ch6*, was identified on chromosome 6 with a peak at 28 cM (approximately 11 Mbp) (Figure 3D). *qRRD_TX2WSE_ch6* had a consensus interval approximately 20 Mbp in size, containing ~1700 genes.

Phenotypic variance explained (PVE) by each QTL was estimated for each run. *qRRV_TX2WSE_ch5* explained 19.3% (SD ± 3.64) of the phenotypic variance for C_t_ value. Using a mixed model, dominance effects of *qRRV_TX2WSE_ch5* were estimated to account for only 1.4–7.3% of the phenotypic variance. PVE was 41.0% (SD ± 1.50) for severity overall and 17.5% (SD ± 6.28) for severity in individual years. *qRRD_TX2WSE_ch1, qRRD_TX2WSE_ch3*, and *qRRD_TX2WSE_ch6* were of smaller effect. *qRRD_TX2WSE_ch1* and *qRRD_TX2WSE_ch3* explained 16% (SD ± 1.70) and 9.3% (SD ± 0.3) of the phenotypic variation in severity overall, respectively, and *qRRD_TX2WSE_ch6* explained 12.8% (SD ± 0.24) of the phenotypic variation for severity in 2021.

QTL genotypes for *qRRV_TX2WSE_ch5* and *qRRD_TX2WSE_ch1* were determined by FlexQTL™ assuming a biallelic model in which *Q* is the allele with a high phenotypic value, and *q* is the allele with a low phenotypic value; thus, a *Q* for C_t_ value indicates a high C_t_ value (corresponding to low virus level), and a *Q* genotype for severity indicates high severity. Theoretically, if only *qRRV_TX2WSE_ch5* is being considered (i.e., assuming no epistasis), an individual assigned a *QQ* genotype for C_t_ value should be *qq* for severity (and vice versa) if the biallelic assumption of the model is correct and penetrance is complete. For some individuals, this was the case (Table S3). M4-4, the parent of TAMU7-20 and TAMU7-30, was frequently given the *QQ* genotype for C_t_ value and the *qq* genotype for severity; ‘Hiawatha’ and DD were frequently assigned the *QQ* and *qq* genotypes for C_t_ value and severity; and ‘MORchari’ (Sweet Chariot) was frequently assigned the *qq* genotype for C_t_ value and *QQ* genotype for severity. In all runs, parents TAMU7-20 and TAMU7-30 were assigned the *Qq* genotype for both traits, and parent J06-20-14-3 was often assigned the *Qq* genotype. However, the genotypes for ‘Old Blush’, ‘Srdce Europy’, ‘Violette’, ‘Papa Hemeray’, and Swamp Rose EB ARE were inconsistent between C_t_ value and severity, and the genotype for WOB26 was inconsistent between severity overall and severity 2021. In addition, more individuals had the *qq* genotype for severity than expected based on known RRD susceptibility, casting doubt on the accuracy of some QTL genotype assignments based on the assumptions outlined above. 

Fewer individuals could be assigned a QTL genotype for *qRRD_TX2WSE_ch1*; however, DD, ‘Old Blush’, *Rosa wichurana* ‘Basye’s Thornless’, ‘Srdce Europy’, and WOB26 were determined to have the *QQ* genotype, corresponding to higher severity (Table S4). ‘Hiawatha’, the unknown pollen parent of J06-20-14-3, ‘MORchari’ (Sweet Chariot), ‘Violette’, and TAMU7-30 were determined to have the *qq* genotype. J06-20-14-3, ‘Papa Hemeray’, TAMU7-20, and M4-4 were assigned the *Qq* genotype. The QTL genotype assignments for TAMU7-30 and M4-4 were not consistent with the haplotyping results for this QTL. Based on these genotype assignments, several individuals are homozygous for favorable alleles for *qRRV_TX2WSE_ch5* and homozygous for unfavorable alleles for *qRRD_TX2WSE_ch1* (DD, ‘Old Blush’, *Rosa wichurana* ‘Basye’s Thornless’) or vice versa (PP-J14-3 and ‘MORchari’ (Sweet Chariot)). Only founders ‘Hiawatha’ and PP-M4-4 were consistently assigned a *QQ* genotype for C_t_ value and *qq* for severity for both QTL.

### 2.4. Haplotype Analysis

Haplotyping was performed for QTLs with PVE greater than 15%. Seven markers from the vicinity of the *qRRV_TX2WSE_ch5* peak were chosen for haplotyping: chr05_10509448, chr05_10094026, chr05_10415546, chr05_10381834, chr05_10542937, chr05_11080653, and chr05_10130872. These markers span approximately 1 cM in the consensus map and approximately 1 Mbp in genome interval. From these markers, 11 haplotypes were identified. Eight haplotypes segregated in the progeny, though two haplotypes had only one occurrence each (Table 2). The most common haplotypes were haplotypes 5B and 5H. Fourteen markers spanning approximately 14 cM (approximately 12 Mbp) from the *qRRD_TX2WSE_ch1* peak were chosen for haplotyping: chr01_141729, chr01_351332, chr01_4779174, chr01_2626480, chr01_371589, chr01_4779270, chr01_6092504, chr01_2626576, chr01_8407507, chr01_8034881, chr01_12365441, chr01_12454481, chr01_11424367, and chr01_5360670. Twenty-one haplotypes were identified, twelve of which segregated in the progeny (Table 3). The most common haplotypes were 1G, 1K, 1F, and 1H.

**Figure 4:**
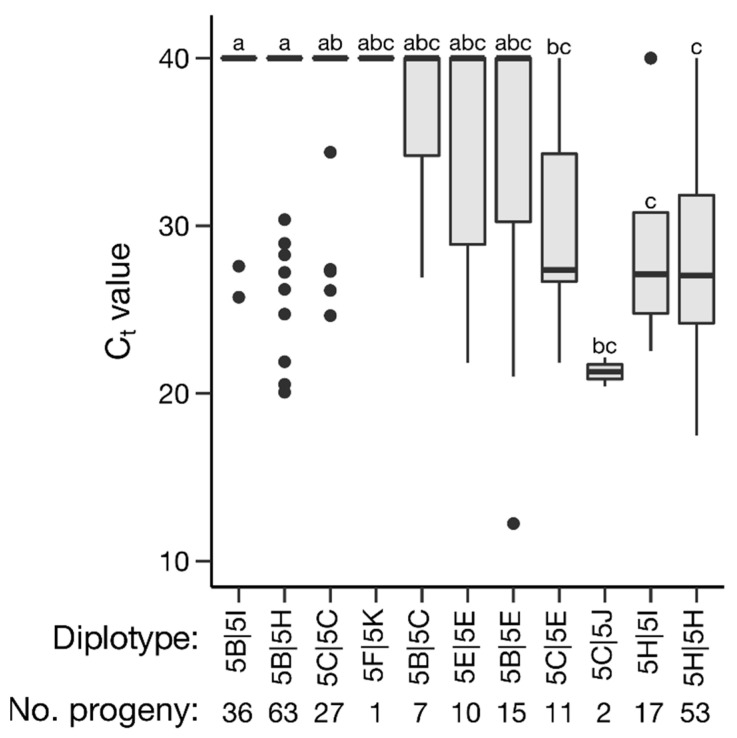
Effects of *qRRV_TX2WSE_ch5* diplotypes on C_t_ value in diploid rose populations. Levels not connected by the same letter are significantly different at *p* < 0.05 (Steel–Dwass nonparametric multiple comparison test).

**Figure 5:**
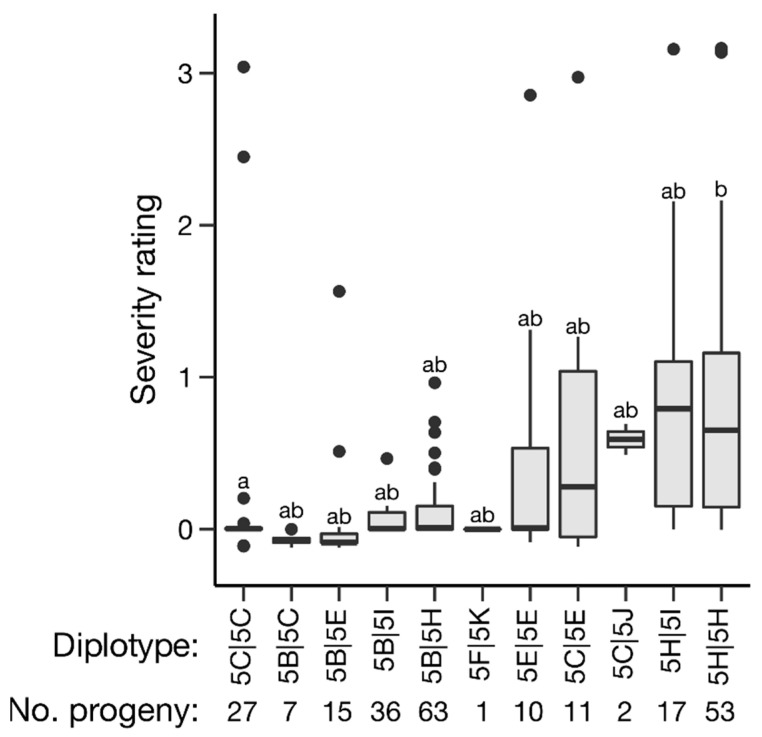
Effects of *qRRV_TX2WSE_ch5* diplotypes on overall severity BLUEs in diploid rose populations. Levels not connected by the same letter are significantly different at *p* < 0.05 (Steel–Dwass nonparametric multiple comparison test).

**Figure 6:**
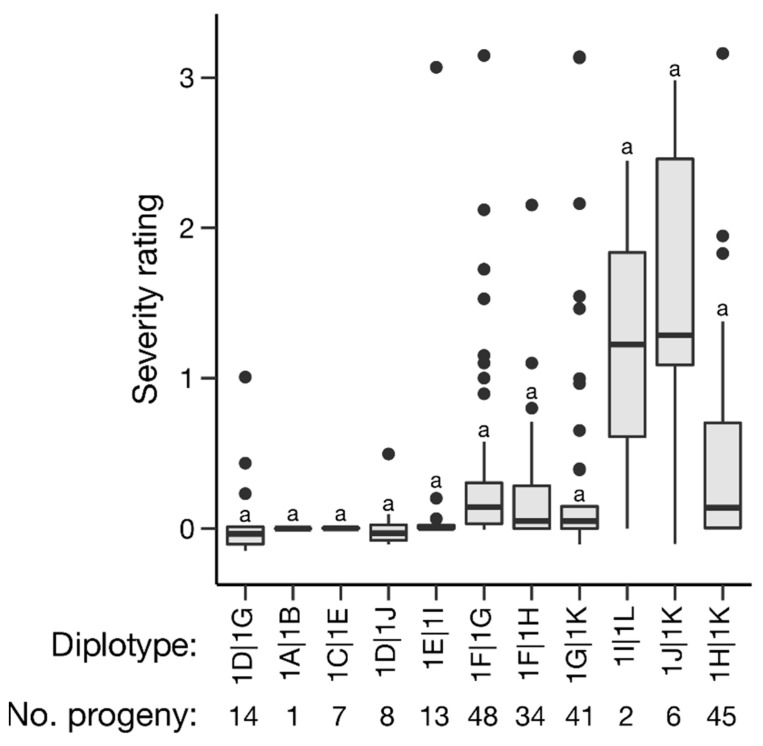
Effects of *qRRD_TX2WSE_ch1* diplotypes on overall severity BLUEs in diploid rose populations. Levels not connected by the same letter are significantly different at *p* < 0.05 (Steel–Dwass nonparametric multiple comparison test).

Haplotype and diplotype effects on C_t_ value and severity overall were estimated; however, effects could only be partially determined. Of the *qRRV_TX2WSE_ch5* haplotypes, 5B, 5H, and 5J had significant effects on C_t_ value (*p* < 0.05); however, haplotype 5J only had two occurrences, making its effect less certain. Haplotype 5B was associated with a higher C_t_ value (lower relative virus quantity) and haplotype 5H with a lower C_t_ value (higher relative virus quantity) (Figure 4, Table 2). Similarly, haplotypes 5B and 5H were associated with reduced and increased severity, respectively (Figure 5, Table 2). Haplotype 5C and 5E also had significant effects on severity, corresponding to a slight decrease and increase in severity, respectively. Of the *qRRD_TX2WSE_ch1* haplotypes, only haplotypes 1D and 1K had significant effects on severity (Figure 6, Table 3). Haplotype 1D was associated with a reduced severity, while 1K was associated with increased severity. A lack of homozygotes hampered the determination of diplotype effects. The effects of compound QTL genotypes could not be determined.

**Figure 7:**
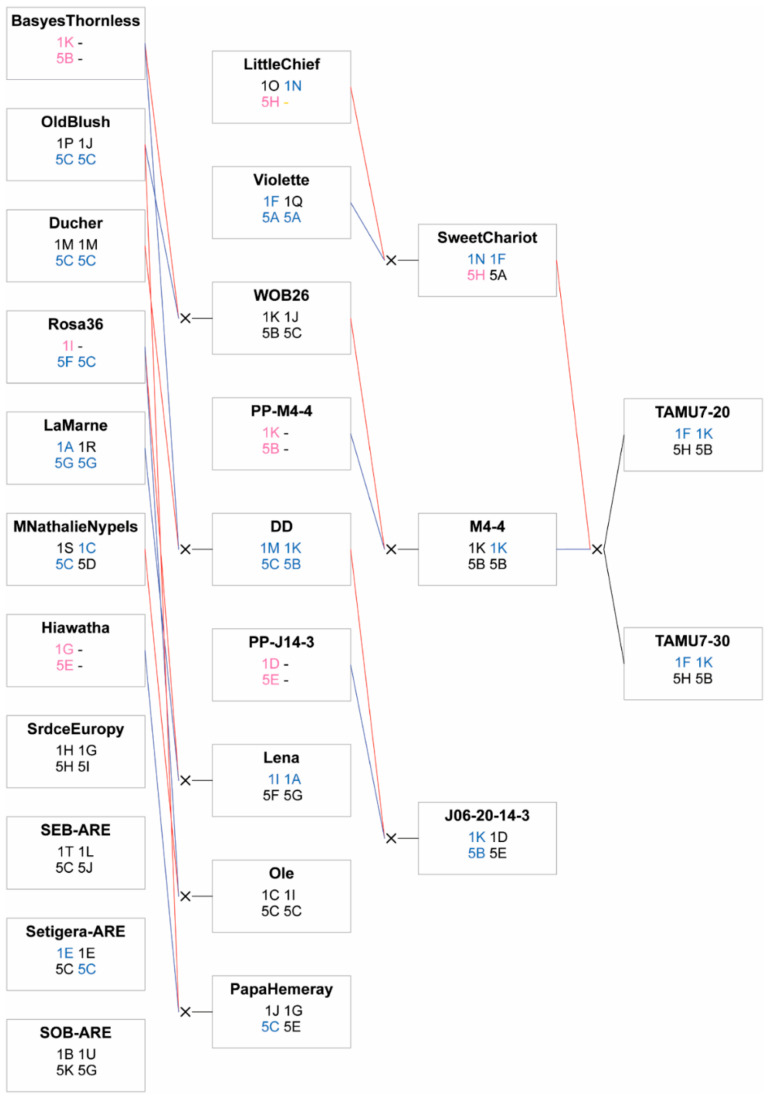
Sources of haplotypes for *qRRV_TX2WSE_ch5* and *qRRD_TX2WSE_ch1* identified in diploid rose populations. Haplotypes are color-coded as follows: black, original score; blue, original score changed by PediHaplotyper; yellow, original score removed by PediHaplotyper; pink, score manually imputed. Red and blue lines indicate female and male parents, respectively. SEB-ARE and SOB-ARE indicate Swamp Rose EB ARE and Swamp Rose OB ARE, respectively.

Pedimap was used to visualize haplotype sources (Figure 7). The presumed source of beneficial haplotype 5B is *Rosa wichurana* ‘Basye’s Thornless’ (genotype unavailable), which contributed the haplotype to progenitors WOB26 and DD, the female parents of M4-4 and J06-20-14-3, respectively. The haplotype 5B passed to TAMU7-20 and TAMU7-30 (progeny of M4-4) may be identical by descent to the ‘Basye’s Thornless’ haplotype; the pollen parent of M4-4 is unknown, but it is likely to be a self-progeny of WOB26. Haplotype 5H, however, appears to come from both ‘MORchari’ (Sweet Chariot) (presumably from ‘Little Chief’) and the pollen parent of the M4-4xSE, T7-20xSE, and T7-30xSE populations. As the identity of this pollen parent is uncertain (see materials and methods), it is unclear if these haplotypes are identical by state or identical by descent. Haplotype 5C, associated with reduced severity, traces to the parental lines ‘Papa Hemeray’, ‘BAIole’ (Ole), *R. setigera* ARE, and Swamp Rose EB ARE, which are unrelated based on available pedigree information. Haplotype 5E traces to parents J06-20-14-3 and ‘Papa Hemeray’; these two copies are likely identical by state based on available pedigree information.

Like haplotype 5B, haplotype 1K, the *qRRD_TX2WSE_ch1* haplotype associated with increased severity, was also traced to *Rosa wichurana* ‘Basye’s Thornless’ and from there to parents J06-20-14-3 and M4-4 (Figure 7). As with haplotype 5B, the copy of haplotype 1K passed to TAMU7-20 and TAMU7-30 from M4-4 may be identical by descent to the ‘Basye’s Thornless’ haplotype. The only source of haplotype 1D, the *qRRD_TX2WSE_ch1* haplotype associated with reduced severity, was J06-20-14-3 through its unknown pollen parent.

## 3. Discussion

The consensus map developed for this study represents an improvement on other high-density linkage maps developed for diploid roses. The length of this map, 759.2 cM, was shorter than the maps of Yan et al. [19] and Li et al. [20], which were 892.2 cM and 1027.43 cM in length, respectively. This map’s density of 1.5 unique positions/cM was also higher than that of the previous maps (0.92 and 0.99 unique positions/cM for Yan et al. and Li et al., respectively), and the maximum distance between markers was also smaller than in these maps. Several recent maps also developed with polymapR, namely Bourke et al. [21] and Zurn et al. [22,23], resulted in shorter, denser maps with smaller maximum gaps than this study’s consensus map. These differences may be due to the use of different germplasm, genotyping platforms, and different population structures.

Three QTLs with a major effect (PVE > 10%) and one QTL of minor effect on RRD C_t_ value and/or severity were identified in a set of inter-related diploid rose populations. *qRRV_TX2WSE_ch5* was detected in all runs for C_t_ value and severity in individual years and across years. The QTL peak is located at approximately 10 Mbp on chromosome 5. The peak of *qRRD_TX2WSE_ch1*, detected in 2019 severity and severity overall, was between 0.3 and 4.8 Mbp on chromosome 1. *qRRD_TX2WSE_ch3*, detected in severity overall, had a peak at 27.8 Mbp on chromosome 3. The peak of *qRRD_TX2WSE_ch6*, which was detected only in 2021 severity, was around 11 Mbp on chromosome 6. While identifying the genes responsible for these QTLs was beyond the scope of this study, all QTL intervals included potential disease-resistance candidates, such as genes coding for NBS-LRR-class disease-resistance proteins and transcription factors (data not shown). Resistance to the mite vector is also a possible breeding strategy, and all QTL intervals contained multiple genes coding for receptor-like protein kinases (data not shown), which have been previously tied to pest resistance in plants [24]. Indeed, a receptor-like protein kinase has been suggested as the causal gene for resistance to an eriophyid mite affecting wheat [25]. Further work would be needed to identify the gene(s) responsible for RRD resistance in these populations.

*qRRV_TX2WSE_ch5* was observed to have minor dominance effects for C_t_ value. For severity, only additive effects were estimated by FlexQTL™, but the heritability estimates suggest the presence of minor non-additive effects as well. It is possible, however, that the dominance effects of C_t_ value may be impacted by its logarithmic nature. A previous study in titratable acidity in apple [26] found that an acidity gene had additive gene action, but it was hypothesized that when acidity was measured with pH (a logarithmic scale) rather than titratable acidity (linear), the gene would appear to have dominant gene action. If this is the case for *qRRV_TX2WSE_ch5*, the use of virus titer rather than C_t_ value may indicate different gene action.

A total of eleven haplotypes for the *qRRV_TX2WSE_ch5* peak were identified in the progeny, parents, and progenitors; eight of these haplotypes segregated in the progeny. Haplotype 5B, associated with a higher C_t_ value (i.e., reduced RRV levels) and reduced severity, could be distinguished from the other 10 haplotypes by marker chr05_10130872 alone; haplotype 5H, associated with lower C_t_ value and increased severity, could be distinguished from the other haplotypes by marker chr05_10509448 alone. Field trial data (not shown) suggest that parents M4-4, J06-20-14-3, TAMU7-20, and TAMU7-30 (carriers of haplotype 5B) have some degree of RRD resistance or reduced susceptibility. It is unclear if the three haplotypes with no effect on severity and/or C_t_ value are truly neutral in effect or if the data were insufficient to determine haplotype effect. Further work is needed to illuminate the effects of these haplotypes, especially haplotype 5C. This haplotype was associated with reduced severity and had no effect on C_t_ value; however, one or more copies of haplotype 5C are carried by individuals susceptible to RRD, such as ‘Old Blush’, which is homozygous for haplotype 5C. 

Two haplotypes from the *qRRD_TX2WSE_ch1* peak were determined to have significant effects on severity overall. Only haplotype 1D, which was distinguishable from the other haplotypes by marker chr01_141729 alone, was associated with reduced severity, but it only occurred in population J14-3xPH. Haplotype 1K, associated with increased severity, appears to derive from *R. wichurana* ‘Basye’s Thornless’, meaning the M4-4/WOB26/’Basye’s Thornless’ lineage carries favorable and unfavorable alleles for RRD severity from *qRRV_TX2WSE_ch5* and *qRRD_TX2WSE_ch1*, respectively. As the effects of *qRRD_TX2WSE_ch1* are small relative to *qRRV_TX2WSE_ch5*, M4-4 is still a promising option for RRD-resistance breeding, as it is homozygous for haplotype 5B. 

QTL genotypes and the effects of the other haplotypes for both *qRRV_TX2WSE_ch5* and *qRRD_TX2WSE_ch1* were not always clear. Most notably, ‘Old Blush’, known to be moderately susceptible to RRD, is homozygous for haplotype 5C and was assigned a conflicting QTL genotype between C_t_ value and severity. Most parents, progenitors, and founders that could not be assigned a consistent *qRRV_TX2WSE_ch5* genotype had one or more copies of haplotype 5C. As in other studies (e.g., Kostick et al. [27], Verma et al. [26]), the presence of multiple *Q* and *q* alleles is likely causing difficulties in accurate assignment of QTL genotypes. Moreover, while the presence of epistasis between severity QTLs could not be established, epistasis could also confuse the assignment of QTL genotypes.

This study was affected by the varying family sizes that were used: family sizes ranged from 1 to 89 progeny. Moreover, representation of parents in the progeny was highly skewed: *R. setigera* ARE, Swamp Rose EB ARE, and Swamp Rose OB ARE had relatively low representation among progeny (*n* = 21, 9, and 1, respectively), while ‘Srdce Europy’ and M4-4 (through TAMU7-20 and TAMU7-30) were well-represented (*n* = 160 and 175, respectively). While small families can be used by FlexQTL™’s pedigree-based analysis, a consequence of using small families was that some haplotypes had very few occurrences and some diplotypes did not occur in the dataset, thereby hampering the determination of some haplotype and diplotype effects.

Future work could involve validating *qRRD_TX2WSE_ch6* and *qRRD_TX2WSE_ch3*, detected in a limited number of runs; using a controlled method of inoculation to ensure adequate disease pressure; and developing larger populations using the parents carrying rare haplotypes or haplotypes of unknown effect to determine their impact on RRD resistance. In particular, the effect of haplotype 5C should be confirmed, as its effects are unclear; yet, it is fairly common in the pedigree of these populations. Further work should be performed to determine if haplotype 5J (from parent Swamp Rose EB ARE) is indeed a source of RRD susceptibility. Breeding efforts ought to focus on individuals known to have haplotype 5B and/or 1D. Thirty progeny from a number of families were identified that carried two *qRRV_TX2WSE_ch5*-favorable haplotypes and no *qRRD_TX2WSE_ch1*-favorable haplotypes. While no progeny carried four favorable haplotypes between *qRRV_TX2WSE_ch5* and *qRRD_TX2WSE_ch1*, 4 and 24 progeny were identified that carried three and two favorable haplotypes between the two loci, respectively. These progeny, all from family J14-3xPH, could be of particular use in pyramiding resistance loci. 

This study marks the first identification of genomic regions associated with RRD resistance in diploid roses. Indeed, while a number of novel emaraviruses have been described in recent years, little is known about genetic resistance to most of these viruses. Notable exceptions are High Plains wheat mosaic virus (HPWMoV), which affects both wheat and maize, and pigeonpea sterility mosaic virus (PPSMV), affecting pigeonpea and close relatives [14]. In the case of HPWMoV, genes conferring virus resistance and resistance to the mite vector have been mapped in maize [28] and wheat (reviewed in [29]), respectively. Multiple QTLs conferring resistance to PPSMV have been identified in pigeonpea, but as in this study, candidate genes have not been identified [30,31]. However, a Kompetitive allele-specific PCR (KASP) assay has recently been designed to aid in the identification of PPSMV-resistant pigeonpea varieties [32]. If the markers in this study that distinguish haplotypes 5B and 1D can be validated, similar marker tests could be deployed in roses to track and manipulate a given QTL and identify progeny likely to have reduced susceptibility to RRD. By identifying four QTLs for RRD resistance, this study lays the groundwork for applying MAS in breeding RRD resistant roses and for a better understanding of the genetic basis for emaravirus resistance in plants.

## 4. Materials and Methods

### 4.1. Plant Material

**Figure 8:**
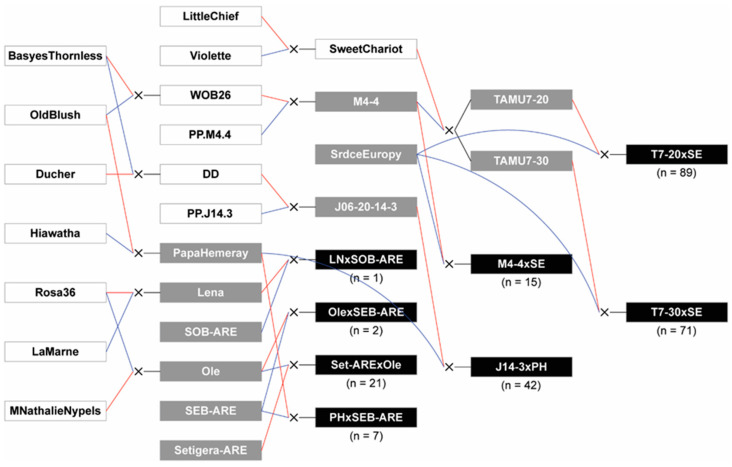
Pedigree of the TX2WSE diploid rose families (indicated in black) used in this study. Progeny numbers indicate the number phenotyped in Crossville, Tennessee for RRD resistance. Direct parents are indicated with gray, and red and blue lines indicate female and male parents, respectively. Founders and progenitors are indicated in white. SEB-ARE and SOB-ARE indicate Swamp Rose EB ARE and Swamp Rose OB ARE, respectively. Figure created with Pedimap v1.2 (Wageningen University and Research, Wageningen, The Netherlands) [33].

Diploid rose crosses were performed in 2015 and 2016 by the Texas A&M Rose Breeding and Genetics Program (College Station, TX, USA) and Weeks Roses (Wasco, CA, USA) to create inter-related populations called TX2WSE that segregated for RRD resistance (Figure 8). Based on the limited resistance data available at the time, ‘Papa Hemeray’, a China-type rose; ‘Srdce Europy’, derived from *R. setigera* and *R. wichurana* Crép.; Swamp Rose, a found rose with an everblooming form (Swamp Rose EB ARE) and a once-blooming form (Swamp Rose OB ARE); and *R. setigera* ARE were considered possible sources of resistance. These were crossed with well-adapted genotypes ‘BAIlena’ (Lena), ‘BAIole’ (Ole), and Texas A&M breeding lines J06-20-14-3, M4-4, TAMU7-20, and TAMU7-30. Eight populations were developed, ranging in size from 1 to 137, for a total of 382 progeny.

### 4.2. Phenotypic Data

As College Station has low RRD pressure, the populations were planted in Crossville, TN, for phenotyping. Due to plant availability and space constraints, only 248 of the genotypes were phenotyped for RRD (population numbers reflected in Figure 6). In 2018, progeny were planted at the University of Tennessee AgResearch Plateau Research and Education Center (36.013814, −85.127247) in a randomized, complete block design with two replications, where individual plants were the experimental unit. Family J14-3xPH was transplanted as mature plants from a nearby field in the same year. Infected cultivars planted in the same field as well as wild *Rosa multiflora* Thunb. growing nearby provided sources of RRV. To further promote disease, in 2019 and 2020, spread of the mite vector was encouraged by attaching RRV-infected shoots to progeny; this was not done in 2018 due to the small size of the plants nor in 2021, as the disease was considered well-established in the field by that point. 

Populations were visually assessed for RRD from 2019 through 2021. In 2019 and 2020, each plant was rated for presence of rosettes on a scale of 0–3, where 0 = no rosettes, 1 = one rosette, 2 = two rosettes, and 3 = three or more rosettes. Furthermore, plants with symptoms were given a severity score on a scale of 0–3, where 0 = no symptoms, 1 = one shoot with symptoms, 2 = two shoots with symptoms, and 3 = three or more shoots with symptoms. In 2021, a 0–5 scale was used such that, for rosettes, 4 = four rosettes, and 5 = five or more rosettes, and for severity, 0 = no symptoms, 1 = <10% of plant symptomatic, 2 = <25% of plant symptomatic, 3 = <50% of plant symptomatic, 4 = <75% of plant symptomatic, and 5 = >75% of plant symptomatic. Data on a 0–5 scale were rescaled to a 0–3 scale for ease of comparison between years. As some rosettes were likely removed by a routine pruning in the spring of 2020 and 2021, the visible symptoms data from 2020 and 2021 were curated such that if a plant had a rosettes/severity score greater than zero in 2019 but a score of zero in 2020 or 2021, we assumed the later data for that plant were questionable and treated them as missing data.

All genotypes (replicates pooled) were tested in 2019 for virus presence using the qRT-PCR method of Dobhal et al. [34]. Only genotypes that were RRV-negative were tested in subsequent years. The resulting cycle threshold (C_t_) values were used as a proxy for RRV levels as other virus quantification methods were not widely available at the time. Samples negative for RRV were assigned a C_t_ value of 40. To combine the three years of RRV data, a single C_t_ value was generated using the lowest C_t_ value for a genotype from the three years.

Data summary and necessary statistical analyses were performed in R v4.0.3 [35]. Correlations between traits were calculated with Spearman’s rank-order test in the R package PerformanceAnalytics [36]. Best linear unbiased estimators (BLUEs) for rosette and severity scores per year and across years were generated in ASReml^®^ v4.1.0 [37] using the linear mixed model

$$P_{ijklm}=\mu +G_{i\left(jk\right)}+F_{j}+M_{k}+E_{l}+FE_{jl}+ME_{kl}+GE_{i\left(jk\right)l}+B_{m\left(l\right)}+\varepsilon_{ijklm}$$

in which $P_{ijklm}$ is the phenotypic value of genotype i at environment *l*; μ is the overall mean; $G_{i\left(jk\right)}$ is the fixed effect of genotype *i* nested in female parent *j* and male parent *k*; $F_{j}$ and $M_{k}$ are the fixed effects of the female parent *j* and male parent *k*, respectively; $E_{l}$ is the random effect of environment *l* (year); $FE_{jl}$ and $ME_{kl}$ are the random interactions of female parent *j* and male parent *k* with environment *l*, respectively; $GE_{i\left(jk\right)l}$ is the random interaction of genotype *i* nested in female parent *j* and male parent *k* with environment *l*; $B_{m\left(l\right)}$ is the random effect of block m nested in environment *l*; and $\varepsilon_{ijklm}$ is the random residual error. 

A model with all effects random was performed in ASReml^®^ using the restricted maximum likelihood method to permit estimation of heritability of rosettes and severity:

$$P_{ilm}=\mu +G_{i}+E_{l}+GE_{il}+B_{m\left(l\right)}+\varepsilon_{ilm}$$

in which $P_{ilm}$ is the phenotypic value of genotype *i* at environment *l*; μ is the overall mean; $G_{i}$ is the random effect of genotype *i*; $E_{l}$ is the random effect of environment *l* (year); $GE_{il}$ is the random interaction of genotype *i* with environment *l*; $B_{m\left(l\right)}$ is the random effect of block m nested in environment *l*; and $\varepsilon_{ilm}$ is the random residual error. Broad-sense heritability (*H^2^*) was estimated from the variance components with the formula

$$H^{2}=\frac{\sigma_{G}^{2}}{\sigma_{G}^{2}+\sigma_{GE}^{2}+\sigma_{\varepsilon }^{2}}$$

in which $\sigma_{G}^{2}$ is the variance of the genotype; $\sigma_{GE}^{2}$ is the variance of genotype x environment; and $\sigma_{\varepsilon }^{2}$ is the residual error variance. Heritability was not estimated for C_t_ value due to the pooling of replicates.

### 4.3. DNA Extraction and SNP Genotyping

Genomic DNA extraction was performed via the CTAB method as described in Yan et al. [19]. Genotyping by sequencing (GBS) was performed using the digital genotyping procedure described in Morishige et al. [38] and Yan et al. [19] with the restriction enzyme *Ngo*MIV. After ligation of a barcoded adapter, samples were sheared via sonication to fragments of approximately 300 bp. A-tailed and T-tailed adapters were added, and PCR was performed to amplify fragments with both adapters. A final PCR was performed to incorporate Illumina bridge amplification sequences.

Single-end sequencing was performed on an Illumina HiSeq 2500 with Illumina protocols (Illumina, San Diego, CA, USA). Only reads with a full match to the barcode and to the partial *Ngo*MIV restriction site were retained. Single-nucleotide polymorphisms (SNPs) were called using the CLC Genomics Workbench v11.0.1 (QIAGEN, Valencia, CA, USA) using the protocol outlined in Yan et al. [19] except that instead of a *Fragaria* genome as a reference the *Rosa chinensis* v1.0 genome [17] was used as a reference. Reads that did not align to the reference genome or aligned at multiple locations were excluded. SNPs were grouped into bins based on their proximity to *Ngo*MIV cut sites in the reference genome and named based on their physical position in the rose genome. 

An examination of the parental genotypes revealed that ‘Srdce Europy’ as genotyped did not agree with progeny genotypes well, perhaps due to plant misidentification, variety heterogeneity, or poor genotyping. Therefore, the ‘Srdce Europy’ genotype was imputed via custom scripts that identified loci in the ‘Srdce Europy’-derived populations where an allele was segregating, but the maternal parent was homozygous; the paternal parent was assumed to be heterozygous at these loci and homozygous otherwise. Since this process results in heterozygous x heterozygous loci as well as some null allele containing crosses misidentified as heterozygous x homozygous, loci with more than 20% homozygous minor allele observations were removed. Ambiguous markers were removed. Progeny with an excessively high number of double recombination events were also removed as part of this process.

### 4.4. Linkage Map Development

Linkage maps were developed for populations J14-3xPH (137 genotypes), T7-20xSE (94 genotypes), and T7-30xSE (82 genotypes). Prior to linkage mapping, SNPs were filtered via a custom script to eliminate low-quality markers. Markers were removed if they could not be mapped to a chromosome of the reference genome, had a high proportion of deletion alleles, were nonbiallelic, had >10% missing data, or if parents were not genotyped for that marker. Markers were also removed for high levels of segregation distortion (chi-square test, *p ≤* 0.0005) with the exception of some marker classes (paternal, maternal, or both) on chromosomes 2, 3, and 6. Markers were then filtered in PLINK v1.9 [39,40] to zero Mendelian-inconsistent errors (MIEs) per population, and markers with >5% MIEs were removed. To reduce complexity, the data for each population were reduced to one marker of each marker class per restriction-enzyme bin, prioritizing the retention of markers common between populations. SNP calls were then converted to dosage via a custom script.

Population maps were developed in the R package polymapR v1.1.1 [41], which was designed for use in polyploids but can also be used for diploids. Simplex x nulliplex markers (equivalent to Aa x aa) in coupling phase were used to identify homologs, and other marker types were assigned to homologs based on their linkage to these markers. Due to a possible translocation between chromosomes 3 and 6 in J14-3xPH, these chromosomes were mapped separately. The Haldane function in MDSMap [42] within polymapR was used to construct maps. Markers that mapped to a different linkage group than their physical position indicated were removed, as were markers with a high nearest-neighbor stress and markers that mapped far from their physical position. Population maps were compared and summarized with the R Shiny application Genetic Map Comparator [43]. 

The consensus map was developed with the R package LPmerge [44] as implemented in the R package Mapfuser [45]. To reduce computation time, the population maps were thinned to one marker every 0.5 cM before consensus map development. The best map was chosen based on the lowest root mean square error, map length, and overall quality. As collinearity with the rose genome was an assumption of the ‘Srdce Europy’ imputation process, correlation of this map with the rose genome was not estimated.

### 4.5. QTL Analysis

Prior to the QTL analysis, further data curation was required. When the inter-related populations were considered together, and progenitor and founder data were added, more MIEs became apparent, and these were zeroed manually. Double recombinations, defined as two recombinations within 10 cM, were identified using FlexQTL™ (Wageningen University and Research, Wageningen, The Netherlands), Microsoft Excel^®^ (Microsoft Corporation, Redmond, WA, USA), and Pedimap v.1.2 (Wageningen University and Research, Wageningen, The Netherlands) [33]. Calls involved in double recombinations were zeroed in parents/progenitors or progeny as needed. Finally, markers missing >50% of their data or that had a >5% increase in missing data were removed entirely. After these steps, 1553 markers remained for further use.

The QTL analyses were performed in FlexQTL™. C_t_ value, per-year BLUEs of severity, and across-years BLUEs of severity were used as phenotypic data; as number of rosettes and RRD severity were highly correlated (ρ > 0.99, *p* < 0.05), and initial runs indicated that rosettes did not provide additional information in the QTL analysis, the trait was not investigated further. Initial runs performed with a mixed model (including both additive and dominance effects) indicated the presence of small dominance effects for both C_t_ value and severity; therefore, an additive model was attempted. Based on both trace sample plots and number of cycles required to reach convergence, a mixed model was deemed most appropriate for C_t_ value, and an additive model was determined to be the best fit for severity (Table S5). The Markov chain lengths ranged from 100,000 to 187,000 to attain convergence (effective chain size ≥ 100). Any singletons (double recombinations around a single marker) remaining after curation were deleted by FlexQTL™ due to the likelihood of genotyping error with GBS. Two different values for the prior distributions (1 or 5, with the former being the minimum allowed by FlexQTL™ and the latter being a frequently used value) were used, with two runs performed for each value to ensure robustness of results. 

Two times the natural log of the Bayes factor (2lnBF) was used to determine presence and strength of QTLs, with 2lnBF > 2 indicating positive evidence for a QTL, 2lnBF > 5 indicating strong evidence, and 2lnBF > 10 indicating decisive evidence [18,46]. The QTL interval was defined as consecutive 2 cM bins with 2lnBF > 2, with the outermost positions of the bins taken as the start and end of the QTL interval. The mode of the QTL was used as the QTL peak. A QTL was considered stable if it was detected by >50% of the runs for the trait. MapChart v2.32 (Wageningen University and Research, Wageningen, The Netherlands) [47] was used to visualize stable QTL, and QTL were named following the QTL naming conventions of the Genome Database for Rosaceae [48].

Following Rawandoozi et al. [49], phenotypic variance explained by additive effects (*PVE_add_*), phenotypic variance explained by dominance effects (*PVE_dom_*), and total phenotypic variance explained by a QTL (*PVE_total_*) were calculated as

$$PVE_{add}=\frac{\sigma_{A\left(qtl\right)}^{2}}{\sigma_{P}^{2}}\times 100$$

$$PVE_{dom}=\frac{\sigma_{D\left(qtl\right)}^{2}}{\sigma_{P}^{2}}\times 100$$

$$PVE_{total}=\frac{\sigma_{A\left(qtl\right)}^{2}+\sigma_{D\left(qtl\right)}^{2}}{\sigma_{P}^{2}}\times 100$$

where $\sigma_{A\left(qtl\right)}^{2}$ and $\sigma_{D\left(qtl\right)}^{2}$ are the additive and dominance effects of a QTL, and $\sigma_{P}^{2}$ is the phenotypic variance of the trait, all of which are estimated by FlexQTL™. Furthermore, heritability was calculated as

$$H^{2}=\frac{\sigma_{trait}^{2}}{\sigma_{P}^{2}}$$

where $\sigma_{trait}^{2}$ is the phenotypic variance minus the residual variance (estimated by FlexQTL™), giving the narrow-sense or broad-sense heritability for an additive or mixed model analysis, respectively.

FlexQTL™ was also used to assign QTL genotypes to parents, progenitors, and founders. A biallelic model was assumed in which *Q* and *q* correspond to a high and low phenotypic value, respectively.

### 4.6. Haplotype Analysis

Haplotyping was performed with FlexQTL™ and the R package PediHaplotyper [50]. The effect of presence or absence of a haplotype on phenotypes was examined with the Kruskal–Wallis test by ranks (*p* < 0.05) in R, and diplotype effects were tested with the Steel–Dwass nonparametric multiple comparisons test (*p* < 0.05) using the R package PMCMRplus [51]. For testing of diplotype effects, partial diplotypes were made missing. Similar to Kostick et al. [27], interactions between QTLs were tested by grouping progeny based on the presence or absence of increased-severity haplotypes across QTLs, then by the number of reduced-severity haplotypes across QTLs (0, 1, 2, or ≥3), for a theoretical total of eight groups. Differences between these groups were tested with the Kruskal–Wallis test by ranks and Steel–Dwass nonparametric multiple comparisons test. 

## Figures and Tables

**Table 1:** Summary of FlexQTL™ runs for RRD severity and C_t_ value in diploid rose populations.

Chr	Trait ^a^	Year	Prior ^b^	Run	BF_(1 vs. 0)_^c^	BF_(2 vs. 1)_^c^	Position (cM) ^d^	Physical Position (Mbp)	PVE (%) ^e^
1	Severity	2019	1	1	3.1	0.2	14 (6–26)	0.4–27.2	15.7
1	Severity	2019	5	2	2.2	0.5	8 (6–28)	0.3–27.2	13.0
1	Severity	Overall	1	1	4	−0.2	10 (4–24)	0.1–27.2	18.1
1	Severity	Overall	1	2	4.2	0.5	8 (4–26)	0.1–27.2	17.0
1	Severity	Overall	5	1	3.1	0.2	16 (4–20)	0.1–12.5	14.0
1	Severity	Overall	5	2	2.9	0.5	12 (4–22)	0.1–12.5	14.2
3	Severity	Overall	1	1	2.2	−0.1	18 (16–30)	9.6–34.3	9.1
3	Severity	Overall	1	2	2	−0.4	18 (16–30)	9.6–34.3	9.7
3	Severity	Overall	5	1	2.1	0.2	18 (16–26)	0.6–33.6	9.1
5	Ctval	Overall	1	1	10.9	1.2	50 (32–60)	5.0–23.5	22.1
5	Ctval	Overall	1	2	11.2	1.7	50 (34–64)	5.0–27.0	23.7
5	Ctval	Overall	5	1	9.9	1.3	49 (34–54)	5.0–18.3	16.3
5	Ctval	Overall	5	2	9.2	1	49 (34–52)	5.0–14.1	15.2
5	Severity	Overall	1	1	31	0.6	49 (42–52)	6.8–14.1	43.2
5	Severity	Overall	1	2	31	0.4	49 (36–50)	5.3–11.1	40.6
5	Severity	Overall	5	1	26.8	0.7	49 (42–52)	6.8–14.1	39.1
5	Severity	Overall	5	2	26.9	0.6	49 (44–52)	6.8–14.1	41.0
5	Severity	2019	1	1	31	0.2	49 (36–52)	5.3–14.1	27.2
5	Severity	2019	1	2	15.8	−0.1	49 (30–52)	3.9–14.1	27.0
5	Severity	2019	5	1	26.8	0.6	49 (36–52)	5.3–14.1	23.6
5	Severity	2019	5	2	27	0.3	50 (36–52)	5.3–14.1	24.5
5	Severity	2020	1	1	9.6	0.7	71 (30–78)	3.9–47.6	17.9
5	Severity	2020	1	2	9.6	0.9	72 (30–74)	3.9–39.5	17.9
5	Severity	2020	5	1	9.8	1.2	70 (54–74)	12.3–39.5	14.3
5	Severity	2020	5	2	9.7	1.6	68 (56–76)	16.0–47.6	13.1
5	Severity	2021	1	1	7.5	2.2	46 (34–58)	5.0–32.7	11.6
5	Severity	2021	1	2	8.9	2.3	46 (34–66)	5.0–32.7	12.2
5	Severity	2021	5	1	7.9	2.2	46 (40–58)	5.3–23.5	10.1
5	Severity	2021	5	2	7.1	2.4	51 (34–58)	5.0–23.5	10.1
6	Severity	2021	1	1	6.4	0.6	28 (4–34)	0.3–34.4	13.0
6	Severity	2021	1	2	5.9	0.2	28 (6–36)	0.3–34.4	13.1
6	Severity	2021	5	1	6.9	0.1	29 (16–32)	3.2–23.2	12.7
6	Severity	2021	5	2	6.8	0.4	28 (16–32)	3.2–23.2	12.5

^a^ All runs for C_t_ value were performed with a mixed model; all runs for severity were performed with an additive model. ^b^ Prior distribution of either 1 or 5 was used. ^c^ BF, Bayes factor (2lnBF) for a one-versus-zero and two-versus-one QTL models, where BF > 2, 5, 10 indicates positive, strong, and decisive evidence, respectively, for one or two QTL(s). ^d^ Peak position followed by QTL range. ^e^ PVE, phenotypic variance explained by additive or additive + dominance effects of the QTL for additive or mixed models, respectively.

**Table 2:** Haplotypes for *qRRV_TX2WSE_ch5* segregating in diploid rose populations and their effects on rose rosette virus C_t_ values and severity BLUEs. Underscores indicate missing data for that SNP in the haplotype sequence. *p*-values less than 0.05 are indicated in bold.

					C_t_ Value					Severity Overall				
		No. Offspring			Presence		Absence			Presence		Absence		
Haplotype Sequence	ID	Homo ^a^	Het ^b^	Total	$\bar{x}$ ^c^	SD	$\bar{x}$ ^d^	SD	*p*-Value ^e^	$\bar{x}$ ^c^	SD	$\bar{x}$ ^d^	SD	*p*-Value ^e^
AATGCCT	5B	0	121	121	37.8	5.5	33.7	5.5	**<0.0001**	0.09	0.23	0.47	0.79	**<0.0001**
AGTGTCA	5C	27	20	47	36.1	6.3	34.5	7.4	0.12	0.25	0.74	0.39	0.71	**<0.0001**
_GATTAA	5E	10	26	36	33.8	7.9	34.9	7.2	0.37	0.39	0.8	0.37	0.7	**0.01**
AG___AA	5F	0	1	1	40.0	NA	34.8	7.3	0.47	0.00	NA	0.37	0.71	0.38
TGATTAA	5H	53	80	133	32.0	7.6	36.4	6.5	**<0.0001**	0.63	0.84	0.22	0.57	**<0.0001**
AGTGCCA	5I	0	53	53	36.1	6.4	34.6	7.3	0.18	0.31	0.60	0.38	0.72	0.78
AATGTCA	5J	0	2	2	21.3	1.2	34.8	7.2	**0.01**	0.59	0.14	0.37	0.71	0.15
AA___CA	5K	0	1	1	40.0	NA	34.8	7.2	0.47	0.00	NA	0.37	0.71	0.38

^a^ Number of progeny homozygous for the haplotype. ^b^ Number of progeny heterozygous for the haplotype. ^c^ Mean phenotypic value of progeny with haplotype. ^d^ Mean phenotypic value of progeny without haplotype. ^e^ Differences in C_t_ value or severity between genotypes with and without haplotype as determined by a Kruskal–Wallis test by ranks.

**Table 3:** Haplotypes for *qRRD_TX2WSE_ch1* segregating in diploid rose populations and their effects on rose rosette severity BLUEs. Underscores indicate missing data for that SNP in the haplotype sequence. *p*-values less than 0.05 are indicated in bold.

		No. Offspring			Presence		Absence		
Haplotype Sequence	ID	Homo ^a^	Het ^b^	Total	$\bar{x}$ ^c^	SD	$\bar{x}$ ^d^	SD	*p*-Value ^e^
CAGTA_AAG_ATGC	1A	0	1	1	0.00	NA	0.37	0.71	0.40
CAG_A_AAG_AT_C	1B	0	1	1	0.00	NA	0.37	0.71	0.40
CATTATGAG_ATGC	1C	0	7	7	0.00	0.00	0.37	0.72	0.17
GATTGTGGGCCTG_	1D	0	22	22	0.06	0.27	0.38	0.72	**<0.001**
CAGTATAAG_ATGC	1E	0	20	20	0.17	0.68	0.38	0.71	0.17
C_GT_TGGGCATGT	1F	0	82	82	0.32	0.56	0.38	0.74	0.22
CAGTATGGGCATGT	1G	0	103	103	0.33	0.68	0.38	0.72	0.88
CAGAATGAGGATAC	1H	0	79	79	0.36	0.57	0.37	0.74	0.09
CAGTATAGT_ATGC	1I	0	15	15	0.39	0.97	0.37	0.70	0.68
CCGTGTAAGCATGC	1J	0	14	14	0.69	1.08	0.36	0.70	0.76
CCGAAAAATGACAC	1K	0	92	92	0.49	0.80	0.34	0.69	**0.04**
CAGTA_AAGCATGC	1L	0	2	2	1.22	1.73	0.37	0.71	0.60

^a^ Number of progeny homozygous for the haplotype. ^b^ Number of progeny heterozygous for the haplotype. ^c^ Mean phenotypic value of progeny with haplotype. ^d^ Mean phenotypic value of progeny without haplotype. ^e^ Differences in severity between genotypes with and without haplotype as determined by a Kruskal–Wallis test by ranks.

## Data Availability

Datasets used in this study will be available in the Genome Database for Rosaceae (http://www.rosaceae.org accessed on 6 June 2022).

## Supplementary Materials

The following supporting information can be downloaded at: https://www.mdpi.com/article/10.3390/pathogens11060660/s1, Table S1: Mean and standard deviation (SD) of rose rosette disease C_t_ value, rosette score and severity ratings from 2019, 2020, and 2021 for each diploid rose family grown in Crossville, Tennessee; Table S2: Summary of three unthinned diploid rose population maps and the integrated consensus map (ICM); Table S3: QTL genotypes for *qRRV_TX2WSE_ch5* for parents, progenitors, and founders estimated by FlexQTL™; Table S4: QTL genotypes for *qRRD_TX2WSE_ch1* for parents, progenitors, and founders estimated by FlexQTL™; Table S5: Settings used in FlexQTL™ software to conduct QTL analyses and justifications for their use.
